# Optimized Synthetic Correlated Diffusion Imaging for Improving Breast Cancer Tumor Delineation

**DOI:** 10.3390/s24248173

**Published:** 2024-12-21

**Authors:** Chi-en Amy Tai, Alexander Wong

**Affiliations:** Department of Systems Design Engineering, University of Waterloo, Waterloo, ON N2L 3G1, Canada; alexander.wong@uwaterloo.ca

**Keywords:** breast cancer, tumor delineation, MRI, synthetic correlated diffusion imaging, optimization

## Abstract

Breast cancer is a significant cause of death from cancer in women globally, highlighting the need for improved diagnostic imaging to enhance patient outcomes. Accurate tumor identification is essential for diagnosis, treatment, and monitoring, emphasizing the importance of advanced imaging technologies that provide detailed views of tumor characteristics and disease. Recently, a new imaging modality named synthetic correlated diffusion imaging (CDI^s^) has been showing promise for enhanced prostate cancer delineation when compared to existing MRI imaging modalities. In this study, we explore the efficacy of optimizing the correlated diffusion imaging (CDI) protocol to tailor it for breast cancer tumor delineation. More specifically, we optimize the coefficients of the calibrated signal mixing function in the CDI^s^ protocol that controls the contribution of different gradient pulse strengths and timings by maximizing the area under the receiver operating characteristic curve (AUC) across a breast cancer patient cohort. Experiments showed that the optimized CDI^s^ can noticeably increase the delineation of breast cancer tumors by over 0.03 compared to the unoptimized form, as well as providing the highest AUC when compared with gold-standard modalities. These experimental results demonstrate the importance of optimizing the CDI^s^ imaging protocol for specific cancer applications to yield the best diagnostic imaging performance.

## 1. Introduction

Breast cancer is a significant cause of death from cancer in women globally [1], highlighting the need for improved diagnostic imaging to enhance patient outcomes. Accurate tumor identification is essential for diagnosis, treatment, and monitoring [2], emphasizing the importance of advanced imaging technologies that provide detailed views of tumor characteristics and disease [3].

Recently, a new imaging modality named synthetic correlated diffusion imaging (CDI^s^) has been showing promise for enhanced prostate cancer delineation when compared to existing magnetic resonance imaging (MRI) modalities [4]. CDI^s^ is an imaging technique that builds on correlated diffusion imaging (CDI). CDI analyzes the distribution of diffusion in the cancerous tissue, with CDI^s^ extending upon this idea by introducing synthetic signals at different gradient pulse strengths and timings where physical acquisitions are not available. The process begins with multiple native signal acquisitions obtained at different gradient pulse strengths and timings, which are then passed into a signal synthesizer that produces synthetic signals. The native signals are then mixed with the synthetic signals to obtain the CDI^s^ signal. Though CDI^s^ served as a strong indicator for prostate presence in tissue [4], a few challenges exist for realizing CDI^s^ for breast cancer.

As defined in [4], there are two key components for producing CDI^s^: (1) the calibrated signal mixing function and (2) synthetic signal acquisitions, which are mixed with native signal acquisitions. The first component uses ρ, which are coefficients that control the contribution of different gradient pulse strengths and timings to produce the CDI^s^ signal. The second component relies on defining $\hat{S}$, the specific synthetic signals to acquire. However, it is non-trivial which values should be used for ρ and $\hat{S}$ in computing the CDI^s^ signals. Furthermore, different responses are elicited by having a very different pulse sequence setup and mixing as explored in this paper.

In this study, we explore the efficacy of optimizing the CDI^s^ imaging protocol to tailor it for breast cancer tumor delineation. More specifically, we optimize the coefficients of the calibrated signal mixing function in the CDI^s^ protocol that controls the contribution of different gradient pulse strengths and timings by maximizing the area under the receiver operating characteristic curve (AUC) in a cohort of breast cancer patients.

## 2. Related Works

MRI is a non-invasive diagnostic imaging method that uses magnetic fields and radio waves to generate detailed images of body organs and tissues [5,6]. For repeated imaging, MRI is considered a safer alternative to X-ray and computed tomography (CT) scans as it uses non-ionizing radiation as opposed to ionizing radiation [6,7]. In this study, the performance of CDI^s^ is compared to that of two different gold-standard MRI modalities: diffusion-weighted imaging (DWI) and apparent diffusion coefficient (ADC).

### 2.1. Diffusion-Weighted Imaging (DWI)

**Figure 1:**
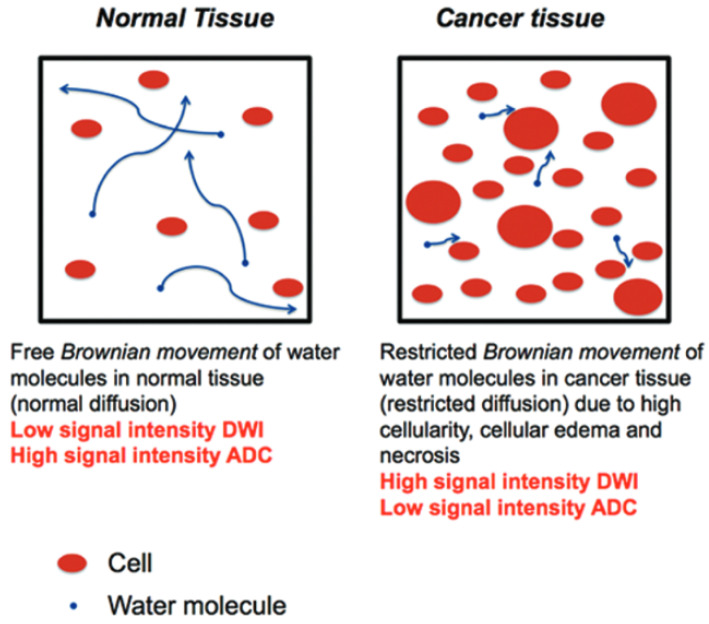
Conceptual illustration of the principle of DWI copied from [13].

Diffusion is the movement of water through tissue and is influenced by tissue density [4]. DWI is a form of MRI that measures the Brownian movement of water molecules within biological tissue [8]. The strength of diffusion sensitivity at which the DWI imaging protocol is configured is typically indicated by the *b*-value, where a *b*-value of 0 indicates no diffusion sensitivity, and diffusion sensitivity increases with higher *b*-values [4,9,10]. Introduced in the late 1980s, DWI has become pivotal in diagnosing and characterizing various medical conditions [11]. It plays a crucial role in the early identification of ischemic strokes, as well as in evaluating tumors and neurodegenerative diseases [12]. Figure 1 highlights the principle of DWI. In normal tissue (left panel of Figure 1), water is able to move freely, which leads to a low DWI signal. On the other hand, in cancer tissue (right panel of Figure 1), the high cellularity, cellular edema, and necrosis restrict the movement of water, which leads to a high DWI signal [13].

### 2.2. Apparent Diffusion Coefficient (ADC)

The quantitative measure derived from DWI is known as the ADC [8]. ADC is obtained by taking the slope of the curve created with the different b values in DWI [8]. Hence, ADC reflects the degree of water diffusion within the tissue, with lower ADC indicating regions with restricted diffusion or potentially cancerous tissue (as seen in Figure 1).

### 2.3. Synthetic Correlated Diffusion Imaging (CDI^s^)

CDI is a newer form of MRI that captures the distribution of water molecules with different degrees of Brownian motion in the tissues within the local volume [14]. The ability to capture the distribution of water molecules with varying degrees of Brownian motion was shown to be effective at delineating between cancerous tissue, which are characterized by wider spreads in the distribution of water molecules with varying degrees of Brownian motion within a local volume, and non-cancerous tissue which are characterized by tighter distributions of water molecules with a similar degree of Brownian motion within a local volume. Synthetic correlated diffusion imaging (CDI^s^) extends upon this idea by introducing synthetic signals at different gradient pulse strengths and timings where physical acquisitions are not available [4]. The methodology for producing CDI^s^ is shown in Figure 2 from [4]. The process begins with multiple native signals obtained at different gradient pulse strengths and timings. These signals are then passed into a signal synthesizer, which produces synthetic signals. The native signals are then mixed with the synthetic signals to obtain a final signal (CDI^s^) [4].

When applied to prostate cancer delineation, Wong et al. [4] showed promising preliminary results for CDI^s^ compared to current MRI techniques. In their extensive study [4], the authors explored the correlation between prostate cancer presence and CDI^s^. Using a cohort of 200 patient cases, the authors assessed the performance of CDI^s^ in delineating PCa against established MRI techniques (T2w, DWI, and dynamic contrast-enhanced imaging (DCE)) [4]. Statistical analyses indicated that CDI^s^ hyperintensity served as a strong indicator of PCa presence, surpassing the delineation capabilities of T2w, DWI, and DCE [4].

### 2.4. Challenges for Harnessing CDI^s^ for Breast Cancer

Though CDI^s^ served as a strong indicator for prostate cancer presence in tissue [4], there exists a few challenges for harnessing CDI^s^ for breast cancer.

As defined in [4], there are two key components for producing CDI^s^: (1) the calibrated signal mixing function and (2) synthetic signal acquisitions, which are mixed with native signal acquisitions. The first component uses ρ, which are coefficients that control the contribution of different gradient pulse strengths and timings to produce the CDI^s^ signal. The second component relies on defining $\hat{S}$, the specific synthetic signals to acquire.

However, the challenge of determining what values to leverage for ρ and $\hat{S}$ is non-trivial as these values can impact the quality of the produced CDI^s^ signal. Selecting optimal parameters by hand is not only labor-intensive but also time-intensive, making it advantageous to identify a strategy for optimizing these parameters for the specific task. Furthermore, since signal mixing is typically done digitally, it is also important that ρ does not have values that are too large, as the mixing function used in obtaining CDI^s^ combines signals multiplicatively, and numerical errors can result if the ρ values being too high.

In [4], where the focus was on prostate cancer, the authors used ρ=1 as their baseline form and also attempted to tune the coefficients ρ using a Nelder–Mead simplex optimization strategy [15]. For their synthetic signal acquisitions, they chose $\{1000\;{s/\mathrm{mm}}^{2}$, …, $7000\;{s/\mathrm{mm}}^{2}\}$ (at $1000\;{s/\mathrm{mm}}^{2}$ intervals) with a native signal acquisition at $50\;{s/\mathrm{mm}}^{2}$. In the context of breast cancer, the initial native signal capture occurs at $0\;{s/\mathrm{mm}}^{2}$, and these signals are not as intense as those in prostate cancer.

### 2.5. Comparison of CDI^s^ to Positron Emission Tomography (PET) Imaging

Positron emission tomography (PET) imaging is another popular imaging modality for radiation oncology [16]. This type of noninvasive imaging leverages high-energy photons and radiotracers to obtain physiological information [17]. In the domain of breast cancer, PET imaging has been shown to aid in breast cancer diagnosis, discrimination, and other important clinical tasks [18,19]. However, CDI^s^ provides a different perspective on tumor tissue characteristics than the metabolic characteristics captured by PET imaging. In addition, CDI^s^ could be more widely accessible since it works on existing MRI machines that are more widely available. Given that MRI machines are generally cheaper than PET, CDI^s^ can be done more frequently but is also notably complementary to PET [20].

## 3. Materials and Methods

### 3.1. Dataset

The pre-treatment patient cohort in the American College of Radiology Imaging Network (ACRIN) study was used as the patient cohort in this study [21,22,23,24]. This dataset is publicly available at [23], and the authors of the public dataset ensured that the patient data were deidentified and anonymous. The timepoint T0 was selected as patients at this stage had not received any neoadjuvant chemotherapy, and thus, the images would be most representative of the ones that pathologists would evaluate to determine SBR grade and decide if the patient should receive neoadjuvant chemotherapy.

The ACRIN 6698/I-SPY2 study contains MRI images across 10 different institutions for patients at four different timepoints in their treatment [21,22,23,24]. The study provides the three main current gold-standard MRI modalities used in clinical practice: DWI acquisitions, T2w acquisitions, and ADC maps. The study also includes detailed annotation metadata (the lesion type, genetic subtype, longest diameter on the MRI (MRLD), the Scarff–Bloom–Richardson (SBR) grade, and the post-treatment breast cancer pathologic complete response (pCR) to neoadjuvant chemotherapy). Manual DWI whole-tumor segmentations were identified using post-contrast DCE subtraction images and then localizing the regions on the ADC map [21,22,23,24].

Patients in the ACRIN 6698/I-SPY2 study were imaged using either a 1.5 or 3.0 Tesla scanner with a dedicated breast radiofrequency coil, and the scanner configuration was static for all images taken for a given patient. Imaging was taken with the patient in the prone position, and both the T2w sequence and DWI (b = 0, 100, 600, 800 s/mm^2^, 3-direction) were performed axially with full bilateral coverage. The pixel spacing for the acquisitions ranged from 0.83 mm to 2.08 mm with a median of 1.29 mm, with both slice thickness and spacing between slices ranging from 4.0 to 5.0 mm with a median of 4.0. ADC maps were then calculated based on the DWI data as a linear fit with ADC values below 0 or voxels below threshold set to 0 to suppress background pixels [21,22,23,24].

Specific MRI acquisition parameters were used to obtain the T2w and DWI images for each patient in the ACRIN 6698/I-SPY2 study. Specifically, the reconstruction matrix was 512 by 512 for T2w and 256 by 256 for DWI with an in-plane resolution of less than or equal to 1.4 mm for T2w and from 1.7 to 2.8 mm for DWI. T2w was performed with active fat-sat recommended while DWI had a fat-suppression parameter value of active fat-sat. The flip angle for both T2w and DWI was 90 degrees with a slice thickness of less than or equal to 4 mm for T2w and between 4 to 5 mm for DWI. The number of slices for both MRI images was variable with complete bilateral coverage for T2w and bilateral coverage with adjustments to keep within a single acquisition for DWI. T2w also had a slice gap of less than or equal to 1.0 mm whereas there was no gap for DWI. The sequence acquisition time was less than or equal to 7 min for T2w images and between 4 and 6 min for DWI with no total post-contrast imaging duration. Four standardized *b*-values were used for DWI acquisitions at 0 s/${\mathrm{mm}}^{2}$, 100 s/${\mathrm{mm}}^{2}$, 600 s/${\mathrm{mm}}^{2}$, and 800 s/${\mathrm{mm}}^{2}$ [21,22,23,24]. More details on the parameter values used to acquire the T2w and DWI images are shown in Table 1 [21,22,23,24].

### 3.2. Data Preprocessing

We use the ACRIN 6698/I-SPY2 study, which contains DWI acquisitions, ADC maps, and DWI whole-tumor segmentations across 10 different institutions for 355 patients at four different timepoints in their treatment [21,22,23,24]. We normalize the MRI images by reducing each MRI volume to 25 slices (the minimum number of slices across all patients) and resizing each image in the volume to 224 × 224. DWI volumes also had an extra dimension for the specific *b*-value. Notably, for comparison, only the DWI images corresponding to a *b*-value of 800 were considered as a previous study showed that only using this *b*-value gained higher performance than using other *b*-values, and the *b*-value of 800 was also more accurate than trying to feed in the data for all the *b*-values for a similar prediction task [25]. All *b*-values are used for the optimization of CDI^s^. One patient had to be removed as they only had three *b*-values instead of four, so 354 patients were used in this study. As the ACRIN 6698/I-SPY2 study does not contain breast masks, breast masks needed to be computed.

For completeness, a computed form of ADC, ADCc, is also calculated based on the specific DWI image. The difference between ADC and ADCc is that ADC is provided in the dataset whereas ADCc is computed manually using a different technique. From the dataset, ADC is calculated as a linear fit to log(S(b))=log(S(0))−ADC∗b with thresholding as defined in [23]. On the other hand, ADCc uses a standard linear regression and linear least-squares formulation with a $R^{2}$ threshold of 0.8 to compute the ADC map.

### 3.3. Experimental Setup

We obtain the optimized synthetic correlated diffusion imaging protocol in this study by leveraging the optimization strategy from [4] with an initial ρ of [1.6160, 1.5209, 1.2006, 0.8362, 1.1630, 0.8666, 1.1424, −0.4635] (showing rounded values for brevity), and $\hat{S}$ values of [50, 1000, 2000, 3000, 4000, 5000, 6000, 7000]. We also added bounds of [−10, 10] for ρ in the optimization to avoid numerical errors when obtaining CDI^s^.

**Figure 3:**
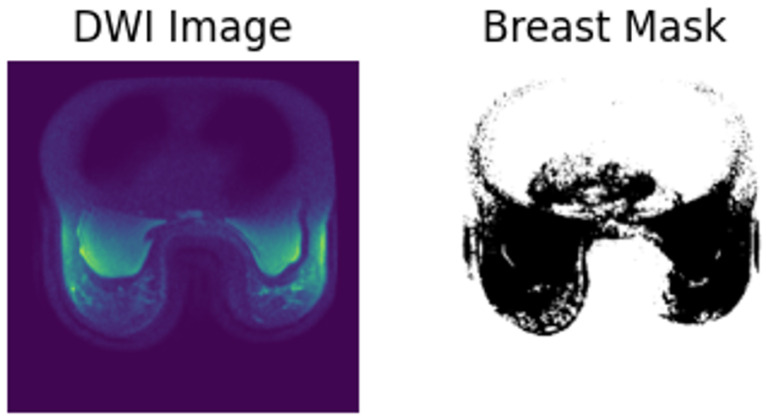
Sample breast mask generated from the DWI image.

As the ACRIN 6698/I-SPY2 study does not contain breast masks, to compute the breast mask, thresholding on the processed DWI images were leveraged along with manual inspection of the resulting breast segmentation mask for quality. An example of the generated mask is provided in Figure 3.

The Nelder–Mead simplex optimization strategy was used to maximize the AUC to improve the ability to delineate between healthy and cancerous breast tissue [15]. Though the Nelder–Mead simplex method is not guaranteed to find the global minimum, it was chosen for its computational efficiency and highly opportunistic behavior [15]. We compare the results from the initial (unoptimized) form against the optimized form for breast cancer. Notably, the AUC value is computed on a voxel basis rather than an average across all patients.

Both qualitative and quantitative evaluations are conducted. Qualitatively, we examine some sample patient images for the various modalities to highlight their visual differences. Histogram analysis is also provided for ADC, ADCc, DWI b = 800, and unoptimized and optimized forms of CDI^s^ values for healthy tissue and cancerous tissue. Quantitatively, AUC values using the gold-standard MRI modalities of ADC, ADCc, and DWI b = 800 are compared with those of the unoptimized and optimized forms of CDI^s^.

To compute statistical significance, we tried to use the DeLong statistical test [26] to gauge whether the AUC improvement was significant between specific modalities. However, given that we are computing AUC at the voxel level, the resulting dataset size of over 1.3 billion values and the needed computation for the DeLong test far exceeded our computational resources. Subsequently, we leverage the Mann–Whitney U test [27] instead as a proxy given our hardware limitations.

## 4. Results

**Figure 4:**
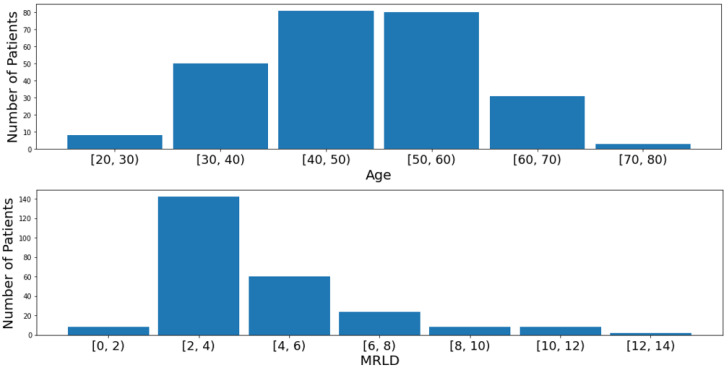
Distribution of the age (**top**) and longest diameter on the MRI (MRLD) in cm (**bottom**) for patients in the cohort.

**Figure 5:**
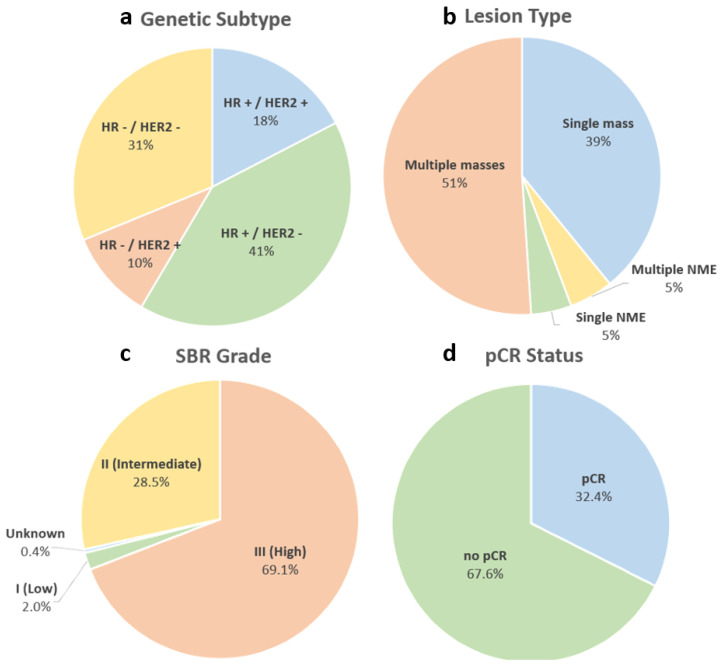
Patient distribution of genetic subtype (**a**), lesion type (**b**), SBR grade (**c**) and pCR status (**d**) in the cohort.

The demographics of the cohort, filtered for patients with non-null pCR values, are shown in Table 2. It can be seen that the White race dominates the cohort, comprising 70.8% of the patients in the cohort, illustrating a severe race bias towards White patients. Additionally, Figure 4 (top) shows that the majority of the patients are between 30 and 70 years old (95.7%), indicating that very young patients (≤29) and very old patients (≥70) could be underrepresented in the cohort. On the other hand, the genetic subtype in the cohort is more fairly distributed, with each subtype represented in at least 10% of the patients, whereas the lesion type is more biased towards multiple masses and a single mass, as seen in Figure 5, upper left and right, respectively. The grade distribution and pCR division for patients filtered with non-null pCR values are shown in the bottom half of Figure 5, indicating an uneven distribution in SBR grade, significantly skewed towards Grade III (High) and shows that more patients with no pCR (67.6%) compared to those who achieved pCR after neoadjuvant chemotherapy (32.4%). In addition, the longest diameter on the MRI (MRLD) is also biased towards the range of 2 to 4 cm with less representation from patients in the other diameter ranges as seen in Figure 4 (bottom).

As seen in Table 3, the optimized ρ values have all changed from the initial values, highlighting the difference from tuning these values. Though the differences may seem small (<10), they have important differences in their resulting values.

**Figure 6:**
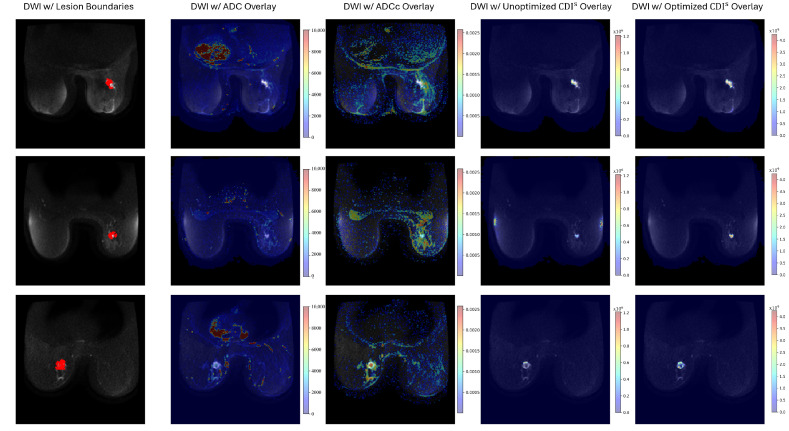
DWI images with overlays of the lesion boundaries, ADC, ADCc, Unoptimized CDI^s^, and Optimized CDI^s^ where the red pixels refer to the lesion for the specific DWI on the far left.

Figure 6 shows the DWI images with overlays of the lesion boundaries, ADC, ADCc, Unoptimized CDI^s^, and Optimized CDI^s^. As seen in Figure 6, CDI^s^ is able to capture the tumor region with the least amount of noise compared to the other modalities. Furthermore, the optimized form of CDI^s^ has a stronger distinction between the tumors and healthy tissues with less noise (e.g., see the second row of Figure 6).

**Figure 7:**
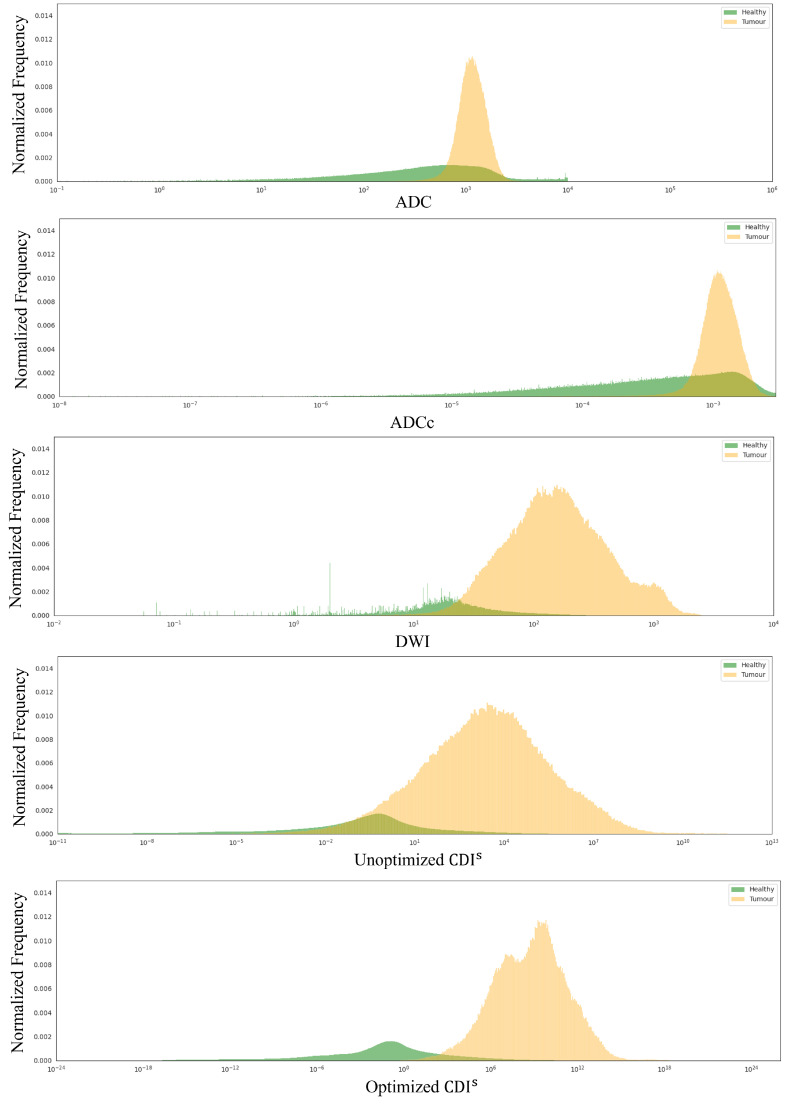
Histogram analysis for ADC, DWI, Unoptimized CDI^s^, and Optimized CDI^s^ values for healthy tissue (green) and tumor tissue (orange) using the DWI breast mask.

To study the distribution of ADC, ADCc, DWI, Unoptimized CDI^s^, and Optimized CDI^s^ for healthy tissue and tumor tissue, histogram analysis was conducted. Figure 7 shows the histogram analysis for the studied modalities for healthy tissue and tumor tissue using the DWI breast mask. As seen, there is a greater separation between healthy tissue and tumor tissue for the Optimized CDI^s^ compared to the other ADC, DWI, and Unoptimized CDI^s^ modalities. Although it may be doubtful that thresholding can identify the entire tumor based on the histograms, a simple threshold of 0.3 would result in an accuracy of 0.76 using the Unoptimized CDI^s^ modality and an accuracy of 0.80 with the Optimized CDI^s^ modality, which is comparable to prior literature studying breast cancer delineation [28].

**Figure 8:**
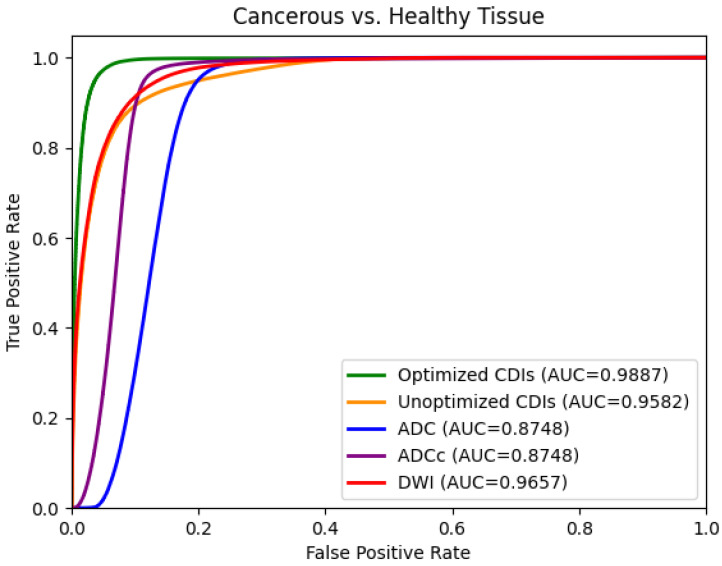
ROC curves comparing the performance of Optimized CDI^s^, Unoptimized CDI^s^, DWI, ADC, and ADCc for delineating cancerous and healthy tissue.

Intuitively, the best AUC value on the processed images is then also achieved by the Optimized CDI^s^ modality, outperforming the best gold-standard modality by 0.023. Notably, the Optimized CDI^s^ modality also achieves AUC values over 0.03 higher than the Unoptimized CDI^s^ value. Figure 8 shows the ROC curves and associated AUC values for the various modalities to separate healthy and tumorous tissue. When comparing the AUC value obtained using the Optimized CDI^s^ modality against the other modalities, we compute a Mann–Whitney U statistic of less than 8.65 for all the modalities and subsequently, an approximate *p*-value of 0 indicating that our results are statistically significant (*p*-value < 0.01 [29]).

## 5. Discussion

Although these results are promising, the optimization of CDI^s^ was conducted using basic threshold-derived breast masks from DWI images and was not verified by experienced radiologists. Moreover, the performance improvement of CDI^s^ over the best gold-standard imaging modality is marginal and could differ for another cohort of patients. Although the Nelder–Mead optimization strategy is widely used, there is still the possibility that the chosen optimization coefficients were not globally optimal and there could be better coefficients to use. Lastly, since tumor masks were not provided for T2w images, the AUC performance could not be computed for the T2w modality, another gold-standard imaging modality, which may be able to better separate healthy and tumor tissue for the breast.

In addition to separating healthy and tumor tissue for breasts, other important clinical breast cancer tasks such as grade classification or predicting pathologic complete response are not studied here. Although promising results were achieved using CDI^s^ for tissue separation, speculation arises about whether using Optimized CDI^s^ would also provide enhancement for other breast cancer clinical support tasks. AUC was used as the key performance in this study as it is critical to the accuracy of tumor detection. In particular, AUC provides a more comprehensive, robust picture of tissue differentiation and different level tradeoffs that radiologists use to visualize tumors.

## 6. Conclusions

CDI^s^ has recently been introduced as a strong indicator for prostate cancer presence in tissue, motivating its application to other cancer domains like breast cancer. However, obtaining CDI^s^ requires two main components: ρ and $\hat{S}$ values to capture the contribution coefficients and specific synthetic signals to acquire, respectively. Experiments showed that the optimized CDI^s^ form can noticeably increase the delineation of breast cancer tumors by over 0.03 compared to the unoptimized form, as well as providing the highest AUC when compared with gold-standard modalities. These experimental results demonstrate the importance of optimizing the CDI^s^ imaging protocol for specific cancer applications to yield the best diagnostic imaging performance. Future work includes the application of CDI^s^ for other cancer domains, such as brain cancer, to enhance clinical support.

## Figures and Tables

**Figure 2:**
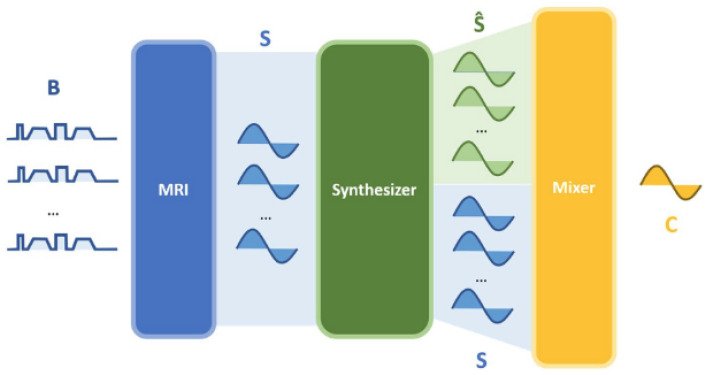
Process to producing CDI^s^ from [4].

**Table 1:** Table of MRI acquisition parameters used to obtain T2w and DWI acquisitions from [21,22,23,24] where (a) refers to the adjustment “up to 400 mm to accommodate for large body habitus if necessary”.

Parameter	T2w	DWI
Sequence type	FSE or STIR	DW SE-EPI
2D or 3D sequence	2D	2D
Slice orientation	Axial or sagittal	Axial
Laterality	Bilateral	Bilateral
Freqency direction	A/P	A/P
Phase direction	R/L (axial)	R/L
	S/I (sagittal)	
FOV–frequency	260–360 mm (axial)	260–360 ${\mathrm{mm}\;}^{\left(a\right)}$
	180–220 mm (sagittal)	
FOV–phase	300–360 mm (axial)	300–360 ${\mathrm{mm}\;}^{\left(a\right)}$
	180–220 mm (sagittal)	
Matrix–frequency (acquired)	256–512	128–192
Matrix–phase (acquired)	≥256	128–192
Reconstruction Matrix	512 × 512	256 × 256
In-plane resolution	≤1.4 mm	1.7–2.8 mm
Fat-suppression	Active fat-sat	Active fat-sat
	recommended	
TR	2000–10,000 ms	≥4000 ms
TE	70–140 ms	Minimum
	(STIR 70 ms)	(50–100 ms)
Echo Train Length	≤16	N/A
TI (STIR sequence)	170 ms (1.5T)	N/A
	230 ms (3.0T)	
Flip Angle	90 degrees	90 degrees
Readout Bandwidth (per pixel)	N/A	N/A
*b* values	N/A	0, 100, 600, 800 s/${\mathrm{mm}}^{2}$
Slice thickness (acquired)	≤4 mm	4–5 mm
# of slices	Variable; complete	Variable; bilateral
	bilateral coverage	coverage; adjust to keep
		w/in single acquisition
Slice Gap	≤1.0 mm	No gap
Parallel imaging factor	≤2	≥2
# of excitations/averages	≤2	≥2
k-space ordering	N/A	N/A
Sequence	≤7 min	4–6 min
acquisition time		(multi-b seq ~5 min)
Total post-contrast	N/A	N/A
imaging duration		

**Table 2:** Summary of race demographics in the cohort.

Race	Percentage
White	70.8%
Black	10.7%
Asian	6.3%
Unknown	11.1%
Multiple Races	0.4%
Native Hawaiian or other Pacific Islander	0.4%
American Indian or Alaska Native	0.4%

**Table 3:** PCa structure optimized parameters for the initial and optimized configurations (Config.).

Config.	Optimized ρ Values							
$\hat{S}$	50	1000	2000	3000	4000	5000	6000	7000
Initial	1.616	1.521	1.201	0.836	1.163	0.867	1.142	−0.464
Optimized	3.517	3.122	1.740	1.00	0.607	0.804	−1.124	−0.655

## Data Availability

The data presented in this study are available in the ACRIN study [23].

## Abbreviations

The following abbreviations are used in this manuscript:

ACRIN	American College of Radiology Imaging Network
ADC	apparent diffusion coefficient
CDI^s^	synthetic correlated diffusion imaging
CT	computed tomography
DCE	dynamic contrast-enhanced imaging
DWI	diffusion-weighted imaging
MRI	magnetic resonance imaging
